# The Systematic Review as a Doctor of Nursing Practice (DNP) Project: A Curricular Innovation in One DNP Program

**DOI:** 10.1111/jan.70141

**Published:** 2025-08-14

**Authors:** Tracy Vitale, William Kernan, Irina Benenson, Rubab Qureshi, Cheryl Holly

**Affiliations:** ^1^ Division of Advanced Nursing Practice Rutgers University, School of Nursing Newark New Jersey USA; ^2^ Division of Nursing Science Rutgers University, School of Nursing Newark New Jersey USA; ^3^ Northeast Institute for Evidence Synthesis and Translation A JBI Center of Excellence Newark New Jersey USA

**Keywords:** advanced practice, doctor of nursing practice, nurse, nursing, nursing education, systematic review

## Abstract

**Aims:**

To advocate for the systematic review as a rigorous, competency‐aligned option for the Doctor of Nursing Practice (DNP) project.

**Methods:**

A descriptive and conceptual analysis was used, drawing on existing literature, historical context, and a case study of a three‐semester curriculum integrating systematic review methodology. Data sources included peer‐reviewed research, professional guidelines, and faculty experience in teaching and mentoring DNP students.

**Results:**

Integrating systematic reviews as DNP projects equips students with competencies in evidence synthesis, critical appraisal, knowledge translation, and project management. A three‐semester scaffolded approach to conducting a systematic review has the potential to foster strong student engagement, build essential skills, and prepare graduates to lead evidence‐based practice change.

**Conclusions:**

Systematic reviews meet DNP project criteria when paired with practice‐focused implementation and evaluation components. This approach offers an alternative where site access, time, or feasibility limits primary data collection, while ensuring methodological rigor and professional relevance.

**Impact:**

Adopting systematic reviews as DNP projects can reduce clinical site burden, expand project opportunities, and strengthen evidence‐based practice capacity in nursing. Broader acceptance and standardization of this model could enhance practice‐based doctoral education globally.

**Patient or Public Contributions:**

No Patient or Public Contribution.

The international presence of doctoral nursing programs has grown significantly since the 1990s, driven by workforce shortages, education needs, and, notably, the expansion of advanced practice roles (Dobrowolska et al. 2021). In the United States, for example, the number of nurses with doctoral degrees has steadily increased over the past decade. The two most common doctoral degrees in nursing, as reported in the 2022 National Nursing Workforce Survey, are the Doctor of Philosophy (PhD) and the Doctor of Nursing Practice (DNP). In 2022, 2.7% of registered nurses held a doctoral degree in nursing compared to 1.6% in 2015. During this time, a shift in the type of doctoral degree awarded to nurses occurred, with more nurses seeking a practice‐focused degree (DNP) than a research‐focused PhD (Smiley et al. 2023).

Several versions of professional practice‐focused doctoral degrees in nursing have existed in the United States since the late 1970s, with curricula that differ from that of the PhD degree. However, these programs were few and did not reflect uniform standards or curricula. Recognising the challenges facing nursing practice and the growing interest in preparing advanced‐level nurses at the doctoral level with a degree that focuses on clinical practice rather than nursing research, in 2004, the American Association of Colleges of Nursing (AACN) released a position statement describing a transition to the Doctor of Nursing Practice (DNP) degree and the development of uniform standards for DNP programs (AACN 2004).

The DNP degree differs from other doctoral‐level degrees in nursing as the focus is on developing effective nursing leaders who can translate evidence into practice (AACN 2004). Some key differences between the PhD and the DNP are that the DNP has less emphasis on theory and less content in research methodology, and more on clinical practice requirements, and emphasis on evidence translation and innovation in health care delivery for the improvement of clinical practice and health outcomes. Further, the requirements for the completion of the two doctoral degrees differ. Whereas the PhD candidate typically develops a research‐driven dissertation, the DNP requirements typically involve a project focused on a clinical practice question designed to improve systems and directly inform practice (Canady 2021).

## 1 Current Expectations

Despite the standardisation of DNP program expectations, a significant amount of variation remained in relation to the final academic product of the DNP degree. In 2015, the AACN clarified recommendations, including those related to the DNP project. In addition to naming the final scholarly product the *DNP Project*, the following are expected to be incorporated into all projects:

(1) Focus on a change that impacts healthcare outcomes through direct or indirect care.

(2) Have a system or population/aggregate focus.

(3) Demonstrate implementation in the appropriate arena or area of practice.

(4) Include a plan for sustainability.

(5) Include an evaluation of processes and/or outcomes (to guide practice and policy).

(6) Provide a foundation for future practice scholarship.

(AACN 2015)

The AACN (2015) goes on to clarify that integrative and systematic reviews alone shall not be considered a DNP project as they fail to provide opportunities to develop and integrate scholarship into practice.

## 2 Current Challenges

Considering the AACN recommendations, it is understandable why most projects take place in either a hospital or an outpatient setting. However, several factors suggest that this structure may not be sustainable. First, the steady growth of DNP enrollment, and therefore DNP projects, creates a potential unanticipated burden on clinical sites. Traditional clinical settings may not always provide the diversity of experiences or specific challenges needed to address emerging healthcare issues. Additionally, while the organisational leadership may be willing to support a student's academic work, limitations may still exist. Current workforce challenges, particularly in healthcare, include doing more with less. The addition of supporting a DNP project on an existing workload may create an undue burden for a unit or site employees, despite the intention of trying to improve practice, outcomes and processes. These challenges may include perceptions of added workload, the need for academic support from content/clinical experts, and coordinated support of ancillary personnel, including quality and information technology departments, research/nursing councils and review boards. Also, for those sites willing to support a DNP project, there may be a limit on how many projects they can support at any given time, further impacting viable options for project sites. If students must identify their own project site, their potential lack of a professional network or work experience may further impact their ability to identify a site. From a site perspective, it is common only to allow those students who are current employees at the site to conduct projects to eliminate added layers of approvals and secure access. As such, innovative thinking is essential when considering the DNP Project.

## 3 The Systematic Review as a DNP Project

The American Association of Colleges of Nursing (AACN) emphasises that DNP projects must extend beyond literature synthesis to include direct applications to practice (AACN 2006). Systematic reviews can meet this mandate as they provide an essential foundation for implementing and evaluating evidence‐based interventions. They also reduce bias and offer a more reliable foundation for clinical decision‐making. Gone is the myth that a systematic review is a sophisticated literature review. A systematic review is a secondary analysis method used to comprehensively collect, evaluate and synthesise all available evidence on a specific topic or research question. The process involves following a structured and predefined protocol to ensure the review is thorough, unbiased and reproducible (see Table 1) (Holly et al. 2023).

**TABLE 1:** Key steps in a systematic review.

**Formulating a Research Question:** Defining a clear and focused question using frameworks like PICO (Population, Intervention, Comparison, Outcome). **Developing a Protocol:** Outlining the methodology, including inclusion and exclusion criteria, search strategy and data extraction methods. **Literature Search:** Conducting a comprehensive search of multiple databases and sources to identify relevant studies. **Study Selection:** Screening and selecting studies based on the inclusion and exclusion criteria. **Quality Assessment:** Evaluating the included studies' quality and risk of bias using standardised tools. **Data Extraction:** Extracting key data from the selected studies for analysis. **Data Synthesis:** Combining the results from the studies, which may include meta‐analysis if the data allows. **Reporting and Interpretation:** Summarising the findings, discussing the implications, and making recommendations for practice, policy or further research.

Systematic reviews have a rich history, evolving significantly in the latter half of the 20th century. The concept gained traction with Archie Cochrane's 1972 book, which emphasised the importance of using evidence from randomised controlled trials in healthcare (Cochrane 1972). This led to the establishment of the Cochrane Collaboration in 1993. In the 1980s, Gene V. Glass coined ‘meta‐analysis’, referring to the statistical synthesis of multiple study results, a key component of systematic reviews (Glass and Smith 1979). By the 1990s, systematic reviews expanded beyond healthcare to fields like education and social policy, with the Campbell Collaboration established in 2000 (EPPI, n.d.).

Systematic reviews have continued to evolve, with methodologies being refined and adapted over time. Today, systematic reviews are integral to evidence‐based practice across various disciplines, synthesising research findings and assessing evidence quality to provide comprehensive overviews of specific topics. They are now a cornerstone of evidence‐based practice in various fields, providing a rigorous and transparent way to synthesise research findings and inform decision‐making. Systematic reviews offer a structured, rigorous approach to analysing existing research on a focused clinical problem. By synthesising high‐quality evidence, DNP students can identify best practices, gaps in current research and opportunities for practice change (Melnyk and Fineout‐Overholt 2019). They consolidate vast research, allowing DNP students to base their interventions on the most reliable evidence available (Higgins et al. 2022), bridging the gap between research and practice by providing clear recommendations for clinical implementation and clinical guidelines (Gough et al. 2017; Straus et al. 2018). DNP projects often focus on quality improvement (QI) in healthcare settings. A systematic review can serve as the basis for designing, implementing and evaluating these initiatives (Titler 2008). Systematic reviews allow DNP students to demonstrate competency in critical appraisal, research literacy, analytical thinking, evidence‐based practice, writing and communication, project management, and team membership (see Table 2).

**TABLE 2:** Competencies inherent in a systematic review as a DNP project.

**Critical Appraisal Skills:** They evaluate and synthesise existing research, assessing the quality and relevance of studies. Additionally, equipping students with critical appraisal skills, using tools like CASP (Critical Appraisal Skills Program) or the JBI frameworks, ensures they can effectively critique any research study. **Research Literacy:** They show an understanding of research methodologies and the ability to navigate academic databases. **Analytical Thinking:** They identify patterns, gaps and inconsistencies in the literature and incorporate insights from various disciplines, enhancing the depth and breadth of the review. **Evidence Synthesis:** They systematically combine and summarise results from multiple research studies to draw more reliable and comprehensive conclusions. It involves evaluating and integrating data from various sources, including randomised controlled trials, observational studies and qualitative research, providing a holistic understanding of a particular topic or question. The aim is to create a clear and cohesive picture that informs evidence‐based practice, policy‐making and further research. Standard evidence synthesis methods include systematic reviews, meta‐analyses and narrative reviews of qualitative evidence. **Knowledge Translation:** They apply research findings to develop recommendations and guidelines. **Writing and Communication:** They effectively communicate their findings and arguments in a clear, structured manner. They clearly and efficiently communicate with team members, ensuring everyone is on the same page. **Project Management:** They plan, organise and complete a comprehensive research project. **Teamwork and Collaboration:** Students work effectively with colleagues, valuing diverse perspectives and expertise. They manage and resolve conflicts that may arise during the collaboration process, and they demonstrate leadership by coordinating tasks, motivating team members and ensuring the project stays on track. **Ethics:** Avoiding plagiarism is a fundamental ethical practice. Students must cite all sources properly, credit the original authors and avoid copying material without proper attribution. They must be transparent about their methods and honest in their reporting. They complete CITI training and IRB approval.

Innovative teaching methods as a foundation for a systematic review implementation model in a school of nursing can make the learning process engaging and effective by transforming the classroom into interactive workshops where students actively participate in the review process through small group exercises, formulating research questions, developing inclusion criteria and practicing data extraction. Real‐world case studies from clinical settings can illustrate the impact of systematic reviews on patient outcomes and policymaking, encouraging students to critique studies and discuss methodologies, and evaluate recommendations (see examples in Table 3).

**TABLE 3:** Examples of the impact of systematic reviews for class discussion.

Lim, C.C., Rutherford, B., Gartner, C. et al. (2024). A systematic review of second‐hand smoking mass media campaigns (2002–2022). *BMC Public Health* 24, 693. https://doi.org/10.1186/s12889‐024‐18222‐5. ○ This review, published in BMC Public Health, summarises the characteristics and outcomes of mass media campaigns on second‐hand smoking (SHS) prevention conducted between 2002 and 2022. The review highlights the effectiveness of these campaigns in raising awareness and reducing SHS exposure, particularly in homes and around children. Gilbert R., Salanti G., Harden M., See S. (2005), Infant sleeping position and the sudden infant death syndrome: systematic review of observational studies and historical review of recommendations from 1940 to 2002, *International Journal of Epidemiology* 34 (4), 874–887, https://doi.org/10.1093/ije/dyi088. ○ This review, published in the International Journal of Epidemiology, systematically examined the associations between infant sleeping positions and SIDS, exploring sources of heterogeneity and comparing findings with published recommendations. The review found a statistically significant increased risk of SIDS for infants sleeping on their front compared to their back, dramatically changing practice in hospitals and at home. Fontaine, G., Vinette, B., Weight, C. et al. (2024). Effects of implementation strategies on nursing practice and patient outcomes: a comprehensive systematic review and meta‐analysis. *Implementation Sci* 19, 68. https://doi.org/10.1186/s13012‐024‐01398‐0. ○ This comprehensive systematic review and meta‐analysis examined the impact of various implementation strategies, such as educational meetings, audit and feedback, and opinion leaders, on promoting evidence‐based nursing practice. The findings demonstrated that these strategies improved clinical practice outcomes and patient outcomes, leading to the widespread adoption of evidence‐based practices in nursing. This review has influenced nursing practice by highlighting the importance of implementing structured strategies to enhance the quality of care and patient outcomes. Olejnik L, Lima JP, Sadeghirad B, et al. (2025). Pharmacologic Management of Acute Pain in Children: A Systematic Review and Network Meta‐Analysis. *JAMA Pediatr*. https://doi.org/10.1001/jamapediatrics.5920. ○ This review and network meta‐analysis examined various pharmacologic treatments for acute pain in paediatric patients, providing valuable insights into the effectiveness and safety of different interventions.

Incorporating technology, such as software tools like Covidence, Rayyan or Open Meta‐analysis, facilitates managing and executing systematic reviews. Virtual discussions, webinars or presentations by experts can provide diverse perspectives. The flipped classroom approach, with pre‐class readings or recorded lectures on the basics of systematic reviews, allows class time for discussions, workshops or practical exercises. Teaching systematic review research to advanced nursing students can seem daunting, but combining interactive, real‐world, and technology‐enhanced strategies can make this essential skill accessible and engaging. By cultivating an environment of collaboration and critical thinking, educators equip their students for successful careers and drive the advancement of evidence transfer, knowledge translation and evidence‐based practice in healthcare.

## 4 Case Study: Modelling the Attainment of Competencies and Signature Assignments When Teaching Systematic Review

We integrate systematic reviews into the Doctor of Nursing Practice (DNP) curriculum over three semesters, equipping students with essential skills and competencies (see Table 2). Each cohort comprises 12 students, divided into small groups of two to three members, who collaborate on a systematic review project. The students choose a primary reviewer/group leader tasked with keeping the group on track and maintaining group cohesiveness (*Competency attained: Teamwork and Collaboration*). Students meet weekly with the course instructor in person or virtually via synchronous video calls. They receive structured guidance through expert‐led lectures, step‐by‐step instructions and iterative assignment feedback. Class sessions are also devoted to discussions, clarifications and student reflections, fostering a collaborative learning environment. We use actual case studies from clinical settings to illustrate the impact of systematic reviews on patient outcomes and policymaking. We encourage students to critique these studies, examine the methodologies used and discuss potential pitfalls and challenges (see examples in Table 3).

In the first semester 3‐credit course, each group develops a systematic review protocol as the *signature assignment* for this course. A series of scaffolded assignments lay the foundation for their projects. These assignments include formulating a clinical question, writing a rationale for the review (background), designing a comprehensive search strategy, outlining detailed methodology, developing a data extraction tool and presenting their protocol to peers and instructors (*Competencies attained: Project Management, Writing and Communication, Research Literacy, Evidence Synthesis, Critical Appraisal, Teamwork and Collaboration*). Lectures by a systematic review methodologist and a medical librarian provide needed support. Structured templates and assignment examples developed by the instructor ensure that students have the tools to develop rigorous and methodologically sound protocols. In this course, students work with the school's IRB liaison to complete an IRB application and complete CITI (Collaborative Institutional Training Initiative) training for IRB (Institutional Review Board), a comprehensive program designed to educate on the ethical and regulatory aspects of conducting research (*Competency attained: Ethics*).

The second semester 2‐credit course focuses on conducting the review as the *signature assignment*. Students work collaboratively to perform comprehensive searches across multiple databases, create PRISMA diagrams, appraise the literature, extract data and synthesise findings using either meta‐analysis or narrative synthesis (*Competencies attained: Analytical Thinking, Critical Appraisal, Research Literacy, Evidence Synthesis, Teamwork and Collaboration*). They receive ongoing mentorship from a research librarian trained in systematic review and a statistician (for meta‐analysis). The institution provides essential resources, including access to multiple research databases, systematic review library services and software tools such as EndNote for reference management, SPSS for statistical analysis and Covidence for streamlining the review process. These resources enable students to conduct systematic reviews efficiently and produce high‐quality work.

The *signature assignment* for the third semester, a 2‐credit course, is knowledge translation. Students finalise their systematic review reports, present their findings by creating posters and develop a knowledge translation project tailored to real‐world clinical applications (*Competency attained: Evidence Synthesis, Knowledge Translation*). These projects may include clinical practice guidelines, patient education materials, policy briefs or training modules for healthcare providers. Working closely with clinical experts and stakeholders, students ensure that their findings are translated into meaningful, evidence‐based interventions that enhance practice. In this course, students close out their IRB approval (*Competency attained: Ethics*).

Student feedback on this structured learning experience has been overwhelmingly positive. Students report gaining skills and confidence in gathering, appraising, synthesising and applying research data to clinical practice. They appreciate the comprehensive course design, expert mentorship and high‐quality instruction throughout the three semesters. Many students highlight that the hands‐on experience of conducting a systematic review and engaging in knowledge translation has strengthened their ability to integrate research into their future practice and lead evidence‐based initiatives that advance healthcare.

## 5 Future Directions

Systematic reviews are essential for Doctor of Nursing Practice (DNP) projects, ensuring both implementation and evaluation in a practice setting. These reviews support practice change by identifying best practices from existing research, making the transfer and adoption of interventions more effective and grounded in strong evidence. After identifying evidence‐based strategies, the student can collaborate with stakeholders to implement a targeted change, such as updating a clinical protocol, launching a pilot program or integrating a new care process. The synthesised knowledge may facilitate the seamless integration of new strategies into existing workflows. The student may initiate this change themselves, or future students may participate in the implementation of the project (legacy project). To fulfil the evaluation component, the student and their team may select appropriate outcome measures (e.g., patient satisfaction, clinical quality indicators, adherence rates) and monitor them over a defined period. The effectiveness of the intervention can be assessed by comparing local outcomes to those reported in the literature (Kranke 2010). This cycle of evidence synthesis, implementation and outcome evaluation ensures that the DNP project not only contributes to the evidence but also improves care delivery in a real‐world setting, meeting the core DNP project requirements.

Emerging technologies can facilitate the systematic review process and aid the DNP student in the completion of the project. Various applications and technologies help simplify the process, and with the growing use of artificial intelligence (AI) in health sciences, the emergence of new technologies is highly probable (Blaizot et al. 2022). AI fields such as text mining, machine learning and natural language processing each offer significant potential for improving specific aspects of the systematic review process. Table 4 presents examples of available technologies and their potential use in the systematic review process.

**TABLE 4:** Potential technologies to support a systematic review as a DNP project.

Stage of SR	Tool name	Purpose & function
Citation Management & Reference	EndNote	Manages references, citations and bibliographies.
	Mendeley	Reference management with PDF annotation and collaboration features.
	Zotero	Free, open‐source tool for managing and sharing research sources.
Literature Search & Screening	Covidence	Streamlines importing, deduplication and screening of studies.
	Rayyan	Supports collaborative blind screening with tagging and filtering.
	EPPI‐Reviewer	Comprehensive tool for managing and analysing review data (search, screen, extract).
Abstract Screening	Abstrackr	Uses machine learning to semi‐automate abstract screening.
Study Prioritisation	ASReview	Uses active learning to prioritise relevant studies for screening.
Data Extraction & Synthesis	DistillerSR	Supports structured data extraction and review tracking.
	SRDR+	Public repository and tool for extracting and managing systematic review data.
Meta‐Analysis	OpenMeta [Analyst]	Conducts meta‐analyses using statistical models (fixed/random effects).
Risk of Bias/Critical Appraisal	RobotReviewer	Uses AI to assess risk of bias in clinical trials automatically.
	ROBINS‐I	Assesses risk of bias in non‐randomised studies.
	Cochrane RoB 2	Evaluates risk of bias in randomised controlled trials.

Systematic reviews centered on existing and emerging key priorities in the field of advanced nursing practice contribute to a better understanding of the nurse's role in the evolving healthcare landscape and in improving patient outcomes. DNP projects that focus on addressing healthcare disparities and identifying opportunities to improve healthcare access among vulnerable populations, for example, have the potential to improve health equity, a key priority of the Institute of Medicine's (IOM) Future of Nursing 2020–2030 Report (Williams and Wakefield 2021). Doctoral projects of this nature align with DNP core curriculum content, allowing students to explore population health concepts, the determinants of health, ethics in nursing practice and the health implications of social factors.

Projects that address the environmental factors that impact healthcare delivery and patient care can improve understanding of the impact of climate change, natural disasters, environmental hazards and the role of sustainable practices (Tiitta et al. 2024). Systematic reviews focused on these topics allow doctoral students to explore epidemiological, ethical and health policy concepts introduced in their core curriculum. Systematic reviews can potentially explore the impact of artificial intelligence (AI) and other technologies, such as telehealth and digital health integration, on advanced nursing practice and patient care (Rony et al. 2024). These projects align with the data and information technology content embedded within the DNP core curriculum.

Additional key priority areas where there is considerable potential for DNP students to engage in systematic reviews as their culminating project include mental health integration, leadership and policy advancement, interprofessional collaboration, nurses' well‐being, clinical decision‐making, healthcare system transformation, chronic disease management and workforce development. Each potential DNP project topic area aligns with core competencies embedded within the DNP curriculum and the key priorities outlined in the Future of Nursing 2020–2030 Report (AACN 2021; Williams and Wakefield 2021).

DNP curriculum revisions should integrate systematic review skills with the core competencies of advanced nursing practice. This can be achieved through curriculum mapping at each stage of the systematic review process (Levin and Suhayda 2018). For example, incorporating systematic review tools into technology training aligns with data and health informatics competency development. Incorporating systematic review content into competency‐based curriculum revision of advanced nursing practice programs can enhance students' critical thinking, evidence‐based practice (EBP) skills and research proficiency.

This model can be easily adapted for non‐US Doctor of Nursing Practice (DNP)‐equivalent programs, enhancing its global reach. Many international nursing doctoral programs share similar goals, such as advancing healthcare policy, improving patient outcomes and integrating research into practice (AERO, n.d.). Adapting systematic reviews for non‐US settings requires consideration of regional healthcare structures, population needs and policy frameworks (WHO 2022). For instance, in Europe, Australia and Asia, systematic reviews inform clinical guidelines, refine education curricula and support evidence‐based decision‐making. By tailoring the review process to local healthcare context, such as disparities in access, workforce composition or disease burden, nursing scholars ensure findings are relevant and actionable (Booth et al. 2019).

## 6 Conclusion

The use of systematic reviews as a doctoral project has meaningful implications for both clinical practice and healthcare policy. This approach offers significant advantages for advancing evidence‐based practice within the nursing profession, while enabling doctoral students to develop essential skills aligned with the advanced practice nurse's role—translating research into practice, enhancing patient outcomes and advancing the nursing discipline. Institutions should consider endorsing systematic reviews as a viable doctoral project option, particularly in practice settings where time, access to participants or feasibility constraints limit primary data collection. Further work is needed to establish standardised guidelines and evaluation criteria for systematic reviews within doctoral curricula, ensuring consistency, academic rigour and alignment with professional practice standards. Conducting a systematic review demands critical thinking, methodological rigour and an in‐depth understanding of the topic. These skills position nurses to become leaders in promoting high‐quality, evidence‐driven care. Because systematic reviews occur at the intersection of research and practice, they are a viable alternative for DNP students when completing their culminating project.

## Author Contributions

All authors made substantial contributions to conception and design, or acquisition of data, or analysis and interpretation of data; involved in drafting the manuscript or revising it critically for important intellectual content; given final approval of the version to be published. Each author participated sufficiently in the work to take public responsibility for appropriate portions of the content; agreed to be accountable for all aspects of the work in ensuring that questions related to the accuracy or integrity of any part of the work are appropriately investigated and resolved.

## Ethics Statement

Data utilised in the submitted manuscript have been lawfully acquired in accordance with The Nagoya Protocol on Access to Genetic Resources and the Fair and Equitable Sharing of Benefits Arising from Their Utilisation to the Convention on Biological Diversity.

## Conflicts of Interest

The authors declare no conflicts of interest.

## Data Availability

The authors have nothing to report.

## References

[jan70141-bib-0001] AERO . n.d. “Evidence‐Based Teaching Practices.” Australian Educational Research Organization.

[jan70141-bib-0002] American Association of Colleges of Nursing . 2004. “AACN Position Statement on the Practice Doctorate in Nursing.” http://www.aacn.nche.edu/DNP/pdf/DNP.pdf.

[jan70141-bib-0003] American Association of Colleges of Nursing . 2006. The Essentials of Doctoral Education for Advanced Nursing Practice. American Association of Colleges of Nursing. DNPEssentials.pdf.

[jan70141-bib-0004] American Association of Colleges of Nursing . 2015. “The Doctor of Nursing Practice: Current Issues and Clarifying Recommendations.” https://www.aacnnursing.org/Portals/42/News/White‐Papers/DNP‐Implementation‐TF‐Report‐8‐15.pdf.

[jan70141-bib-0005] American Association of Colleges of Nursing . 2021. “The Essentials: Core Competencies for Professional Nursing Education.” https://www.aacnnursing.org/Portals/0/PDFs/Publications/Essentials‐2021.pdf. 10.1016/j.profnurs.2025.02.003 40074377

[jan70141-bib-0006] Blaizot, A. , S. K. Veettil , P. Saidoung , et al. 2022. “Using Artificial Intelligence Methods for Systematic Review in Health Sciences: A Systematic Review.” Research Synthesis Methods 13, no. 3: 353–362. 35174972 10.1002/jrsm.1553

[jan70141-bib-0007] Booth, A. , G. Moore , K. Flemming , et al. 2019. “Taking Account of Context in Systematic Reviews and Guidelines Considering a Complexity Perspective.” BMJ Global Health 4, no. Suppl 1: e000840. 10.1136/bmjgh-2018-000840. PMC6350703 30775011

[jan70141-bib-0008] Canady, K. 2021. “Practical and Philosophical Considerations in Choosing the DNP or PhD in Nursing.” Journal of Professional Nursing 37, no. 1: 163–168. 33674087 10.1016/j.profnurs.2020.06.002

[jan70141-bib-0009] Cochrane, A. L. 1972. Effectiveness and Efficiency: Random Reflections on Health Services. Royal Society of Medicine Press.

[jan70141-bib-0010] Dobrowolska, B. , P. Chruściel , A. Pilewska‐Kozak , V. Mianowana , M. Monist , and A. Palese . 2021. “Doctoral Programmes in the Nursing Discipline: A Scoping Review.” BMC Nursing 20: 1–24. 34781935 10.1186/s12912-021-00753-6 PMC8591938

[jan70141-bib-0011] EPPI Centre . n.d. “History of Systematic Reviews.” EPPI Centre.

[jan70141-bib-0012] Glass, G. V. , and M. L. Smith . 1979. “Meta‐Analysis of Research on the Relationship of Class Size and Achievement.” Educational Evaluation and Policy Analysis 1: 2–16.

[jan70141-bib-0013] Gough, D. , S. Oliver , and J. Thomas . 2017. An Introduction to Systematic Reviews. Sage Publications.

[jan70141-bib-0014] Higgins, J. P. T. , J. Thomas , J. Chandler , et al. 2022. Cochrane Handbook for Systematic Reviews of Interventions. Wiley.

[jan70141-bib-0015] Holly, C. , S. Salmond , and M. Saimbert . 2023. Comprehensive Systematic Review for Advanced Nursing Practice. Springer.

[jan70141-bib-0016] Kranke, P. 2010. “Evidence‐Based Practice: How to Perform and Use Systematic Reviews for Clinical Decision‐Making.” European Journal of Anaesthesiology 27, no. 9: 763–772. 10.1097/EJA.0b013e32833a560a. 20523217

[jan70141-bib-0017] Levin, P. F. , and R. Suhayda . 2018. “Transitioning to the DNP: Ensuring Integrity of the Curriculum Through Curriculum Mapping.” Nurse Educator 43, no. 3: 112–114. 28817477 10.1097/NNE.0000000000000431

[jan70141-bib-0018] Melnyk, B. M. , and E. Fineout‐Overholt . 2019. Evidence‐Based Practice in Nursing & Healthcare: A Guide to Best Practice. Lippincott Williams & Wilkins.

[jan70141-bib-0020] Rony, M. K. K. , M. R. Parvin , and S. Ferdousi . 2024. “Advancing Nursing Practice With Artificial Intelligence: Enhancing Preparedness for the Future.” Nursing Open 11, no. 1: 1–9. 10.1002/nop2.2070. PMC10733565 38268252

[jan70141-bib-0021] Smiley, R. A. , R. L. Allgeyer , Y. Shobo , et al. 2023. “The 2022 National Nursing Workforce Survey.” Journal of Nursing Regulation 14, no. 1: S1–S90.37012978

[jan70141-bib-0022] Straus, S. E. , P. Glasziou , W. S. Richardson , and R. B. Haynes . 2018. Evidence‐Based Medicine: How to Practice and Teach It. Elsevier.

[jan70141-bib-0023] Tiitta, I. , F. Cubelo , R. McDermott‐Levy , J. J. K. Jaakkola , and L. Kuosmanen . 2024. “Climate Change Integration in Nursing Education: A Scoping Review.” Nurse Education Today 139: 106210. 10.1016/j.nedt.2024.106210106210. 38643656

[jan70141-bib-0024] Titler, M. G. 2008. “The Evidence for Evidence‐Based Practice Implementation.” In Patient Safety and Quality: An Evidence‐Based Handbook for Nurses, edited by R. G. Hughes , vol. 1, 113–134. Agency for Healthcare Research and Quality. 21328752

[jan70141-bib-0025] WHO . 2022. “Evidence, Policy, Impact: WHO Guide for Evidence‐Informed Decision‐Making.” https://www.who.int/publications/i/item/9789240039872.

[jan70141-bib-0026] Williams, D. R. , and M. K. Wakefield . 2021. “National Academies of Sciences, Engineering, and Medicine; National Academy of Medicine; Committee on the Future of Nursing 2020–2030.” In The Future of Nursing 2020–2030: Charting a Path to Achieve Health Equity, edited by J. L. Flaubert and S. Le Menestrel . National Academies Press. 34524769

